# Multi-sensor, multi-device smart building indoor environmental dataset

**DOI:** 10.1016/j.dib.2023.109392

**Published:** 2023-07-14

**Authors:** Ufuk Erol, Francesco Raimondo, James Pope, Samuel Gunner, Vijay Kumar, Ioannis Mavromatis, Pietro Carnelli, Theodoros Spyridopoulos, Aftab Khan, George Oikonomou

**Affiliations:** a University of Bristol, Bristol, UK; b Toshiba Europe Limited, Bristol Research and Innovation Laboratory, Bristol, UK; c Cardiff University, Cardiff, UK

**Keywords:** Environmental, Internet of Things, Data drift, Sensor, Smart building, Time series dataset

## Abstract

A dataset of sensor measurements is presented. Our dataset contains discrete measurements of 8 IoT devices located in various places in a research lab at the University of Bristol. Nordic nRF52840 DK IoT devices periodically collects environmental data, such as temperature, humidity, pressure, gas, room light intensity, accelerometer; including also a measurement quality indicator. The measurements were taken every 10 seconds over a six-month period between February and September 2022. In addition, we provide Received Signal Strength Indicator (RSSI) of the IoT devices.

The data files are formatted as CSV files. There are various software libraries available to access and read this file format. We provide “README.txt” file which explains the repository and how to use dataset. Each data file is named according to its creation date and, once it reaches a size of 1MB, it is compressed and archived. A new folder is created every week to store all the data files from that week automatically. The dataset can be used for drift detection such as malicious or anomaly detection algorithms. It can also be used for smart building applications like occupation detection. The dataset can be found at https://data.bris.ac.uk/data/dataset/fwlmb11wni392kodtyljkw4n2

**Specifications Table**

**Table:** 

Subject	Engineering, Electrical and Electronic Engineering, Cryptography and Cybersecurity, Embedded Systems, Information Systems
Specific subject area	Smart building, Time series dataset
Type of data	Time series sensor observations from indoor activities of daily work office performed by 28 occupants. The sensor readings are generated in the form of numeric values. Weekly data is stored as CVS files including time(epoch), device IDs, sensor types and measured values. All the CSV files are named with the date and time values which were taken when the files are saved during the measurement period.
How the data were acquired	The data was acquired using several sensors in a smart building/office environment. The sensors were integrated to an IoT Nordic nRF52840 DK board. The following sensors were employed: (1) “ISL29125” Light Sensors: Collects intensity of the light [1]. (2) “MMA8452Q” Accelerometer Sensors [2]. (3) “BME680” Environmental Digital Sensors: Comprise of gas (VOC/ CO₂), pressure, temperature and humidity sensors [3]. The sensors were connected to an IoT device equipped with a microcontroller and radio capabilities. The Message Queuing Telemetry Transport (MQTT) [4] was used as the publish and subscribe communication protocol for gathering data and sending it to a central database server for storage.
Data format	The data consists of raw sensor values formatted either as integer or floating point data types. The raw data includes time, device ID, sensor type and the values only. The device IP addresses are replaced with random indicators such as “A”, “B”, “C”. Furthermore, each data value is timestamped with a Unix epoch (or Unix time or POSIX time or Unix timestamp) value to indicate the time point at which the value was recorded. Each sensor associated with the IoT device is indicated with device ID.
Description of data collection	In total, eight identical severely constrained IoT devices were located in different locations in the office measuring six different values from each sensor every 10 seconds. The data was collected using non-obtrusive environmental sensors. In order to capture different scenarios within the office environment, on each IoT device, 6 different types of sensors were used, namely light, movement(accelerometer), temperature, humidity, gas (VOC/ CO₂) and pressure sensors. In addition to the sensors, we provide Received Signal Strength Indicator (RSSI) values from each device. The IoT devices communicate via radio with an edge device, consisting of an UMBRELLA node [5]. The edge node forwards data to a desktop server to store data. The data was collected continuously over a period of six months.
Data source location	Institution: University of Bristol City: Bristol Country: United Kingdom GPS Coordinates: 51.455643, -2.602358
Data accessibility	Repository name: Multi-sensor, Multi-device Smart Building Indoor Environmental Dataset Data identification number: 10.5523/bris.fwlmb11wni392kodtyljkw4n2 Direct URL to data: https://data.bris.ac.uk/data/dataset/fwlmb11wni392kodtyljkw4n2

## Value of the Data

• The rapid increase in the number of IoT applications has resulted in billions of devices being deployed, producing vast amounts of data. These devices are used for various purposes such as monitoring indoor air quality, estimating occupancy, detecting drift, and planning networks. However, gaining access to real-world data presents a significant challenge due to the reluctance of real-world institutions to disclose it. This limited access makes it difficult to test, standardize, and compare sensor-related technologies. For example, Chimamiwa et al. [6] recently provided a dataset of smart homes over a six-month period, and our proposed dataset has been generated over a similar time frame, with similar sensors, in a working office environment. The continuous monitoring data provided by our dataset is a valuable resource for researchers. Open access to real-world sensor data can benefit the research community, particularly for those who do not have the resources or time to create comprehensive datasets. The availability of such datasets can also speed up the development of algorithms for smart buildings and home automation.
• Gaining a thorough understanding of the true value of a dataset necessitates taking into account the contextual information about the environment and the dataset processing. To this end, we have crafted an openly accessible dataset that has been meticulously collected over a period of six months, leveraging a diverse set of sensors positioned in multiple rooms within a bustling environment. In addition, we have thoughtfully included comprehensive details about the environmental conditions, aimed at providing deeper insights and facilitating the interpretation of the data.
• Open access to raw data from sensors will help advance the development of algorithms for smart office/home environments, such as activity and intrusion detection. Data that has been collected over an extended period of time continuously provides a valuable opportunity to evaluate various machine learning algorithms in areas such as identifying patterns in behavior [7] or detecting anomalies [8].
• An example of how the dataset can be practically used is to test drift detection algorithms designed to identify compromised IoT devices that report false data. This type of manipulation can happen gradually over time and can mislead the state of the environment. To demonstrate this, we assume a device has been hacked and is providing incorrect sensor readings. We focus on gradual manipulation since sudden changes are easier to detect. Additionally, there are natural drifts in the data caused by seasonal changes and abnormal scenarios such as temporary HVAC failures resulting in deviations from ideal indoor temperatures. The dataset can be used to examine both malicious and natural data drifts [13]. Other potential applications of the dataset include occupancy detection, indoor air quality estimation, and evaluating techniques for addressing missing data in time-series data generation.

## 1 Objective

Real-world datasets tend to be non-stationary due to their nature when their distribution alters over time. Environmental changes frequently cause anomalous readings in data in smart building applications and change the trend of the data being streamed. In addition, low-cost hardware standard in environmental sensing and security gaps in IoT networks leaves the streamed data open to malicious attacks.

## 2 Data Description

We provide CSV file versions in a file, named according to timestamps. The file tree can be seen at Table 1. Every week, a new folder is created automatically, to store all the data files of the completed week. Every data file is named using the date of creation and when a file reaches a size of 1MB, it is compressed and archived. Each data file includes 4 columns that represent the time (Unix Epoch) that data was collected, the device ID, sensor type and the measured value. The statistical values of the dataset such as minimum, maximum and standard deviation is shown Table 2.

**Table 1:** Sensor readings of an endnote and the data file structure.

CSV File Content				
Time	DeviceId	Sensor	Value	*File Tree*
1644607900	H	Temperature	24.33499908	*…*
1644607900	H	Humidity	23.83399963	*\—*
1644607900	H	Pressure	102546	*SYNERGIA_Data_Drift*
1644607900	H	Gas	535.276001	*| README.txt*
1644607900	H	Accelerometer	0	*|*
1644607900	H	Light	3736	*\—files_csv*
1644607900	H	MIC	3	*(CSV files)*
1644607900	H	RSSI	83	
1644607901	C	Temperature	20.54500008	
1644607901	C	Humidity	30.24099922	
1644607901	C	Pressure	102557

**Table 2:** Statistical values of the dataset.

Location	Sensor	Minimum	Maximum	Average	Standard Deviation
Room1	Temperature	16.81	45.55	21.95	1.89
	Gas	400.00	16529.76	2290.62	1929.66
	Humidity	8.77	67.40	39.31	8.64
	MIC	1.00	3.00	2.81	0.48
	Pressure	97871.86	103772.37	101251.60	889.11
	Accelerometer	0.00	0.00	0.00	0.00
	Light	0.00	65535.00	1458.95	3623.73

Room2	Temperature	17.26	40.17	22.54	1.73
	Gas	400.00	6350.53	1276.14	860.99
	Humidity	14.74	65.95	38.92	7.97
	MIC	0.00	3.00	2.39	0.89
	Pressure	97869.67	103777.39	101249.54	894.96
	Accelerometer	0.00	0.06	0.00	0.00
	Light	0.00	65535.00	1456.43	3619.48

Room3	Temperature	15.17	32.97	22.39	1.59
	Gas	400.00	10362.51	1271.94	890.63
	Humidity	18.29	61.02	37.86	7.91
	MIC	1.00	3.00	2.49	0.84
	Pressure	97919.25	103792.70	101266.88	898.60
	Accelerometer	0.00	0.70	0.00	0.01
	Light	0.00	65535.00	652.85	1712.27

Room4	Temperature	19.53	29.55	23.21	1.37
	Gas	400.00	12729.53	2584.04	2078.53
	Humidity	17.97	64.71	36.25	7.89
	MIC	0.00	3.00	2.78	0.52
	Pressure	97836.48	103760.66	101261.19	916.18
	Accelerometer	0.00	0.76	0.00	0.03
	Light	0.00	6232.83	834.96	1395.83

**Fig. 1:**
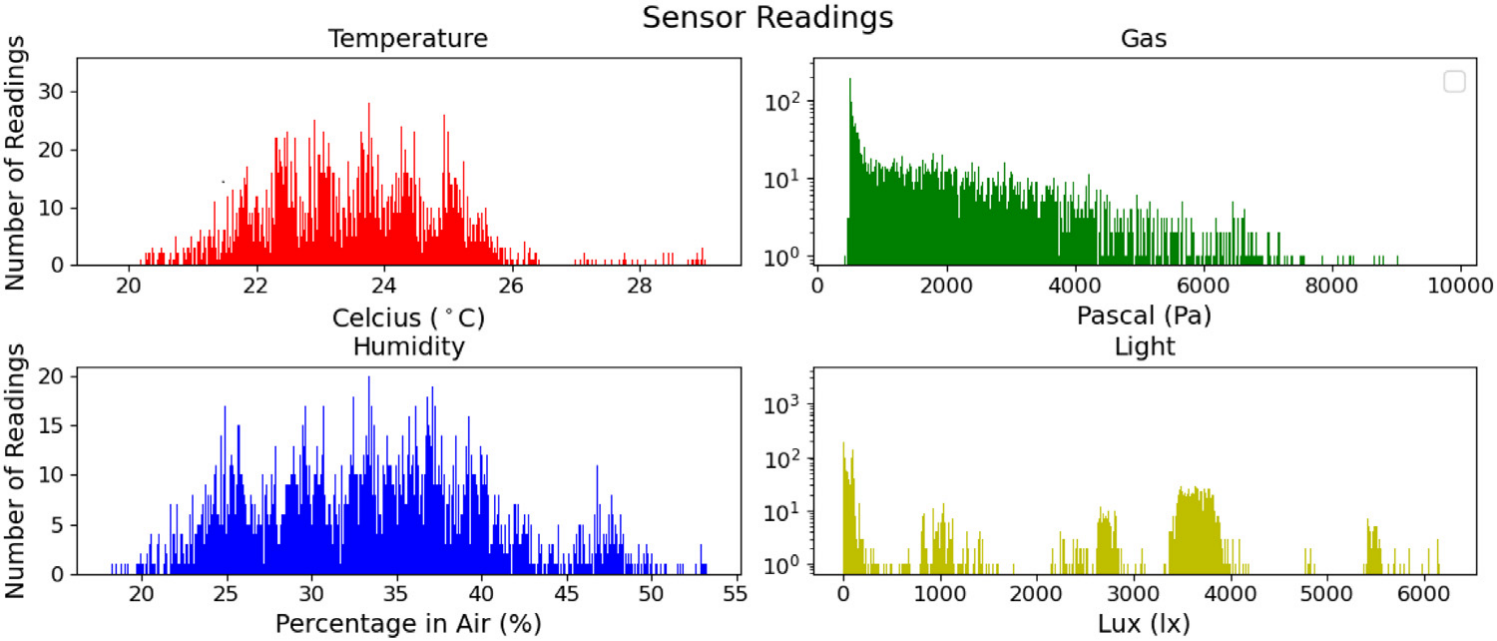
Cumulative distribution of collected data.

**Fig. 2:**
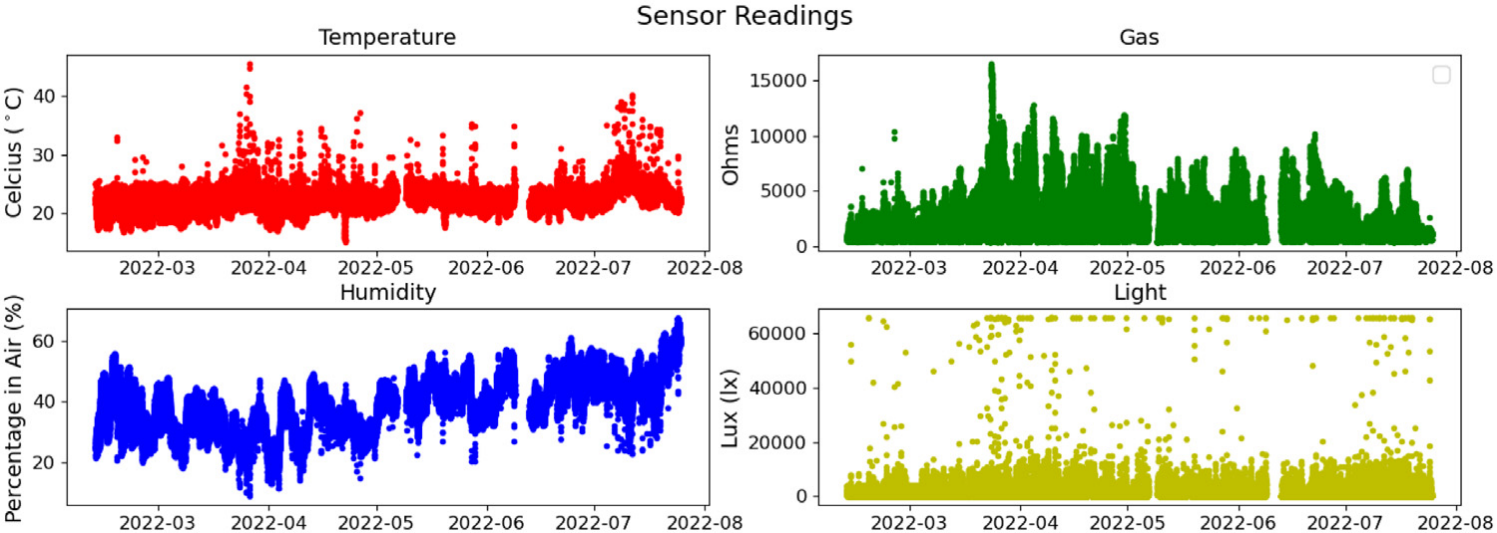
Collected data over six months.

The visualisation of collected data as shown in the Fig. 1 and Fig. 2. The data is collected every 10 seconds for each device and the sensors integrated to them. However, we have some missing data in May and June due to the electricity cut as shown in Fig. 2.

## 3 Experimental Design, Materials and Methods

### 3.1 Experiment overview

**Fig. 3:**
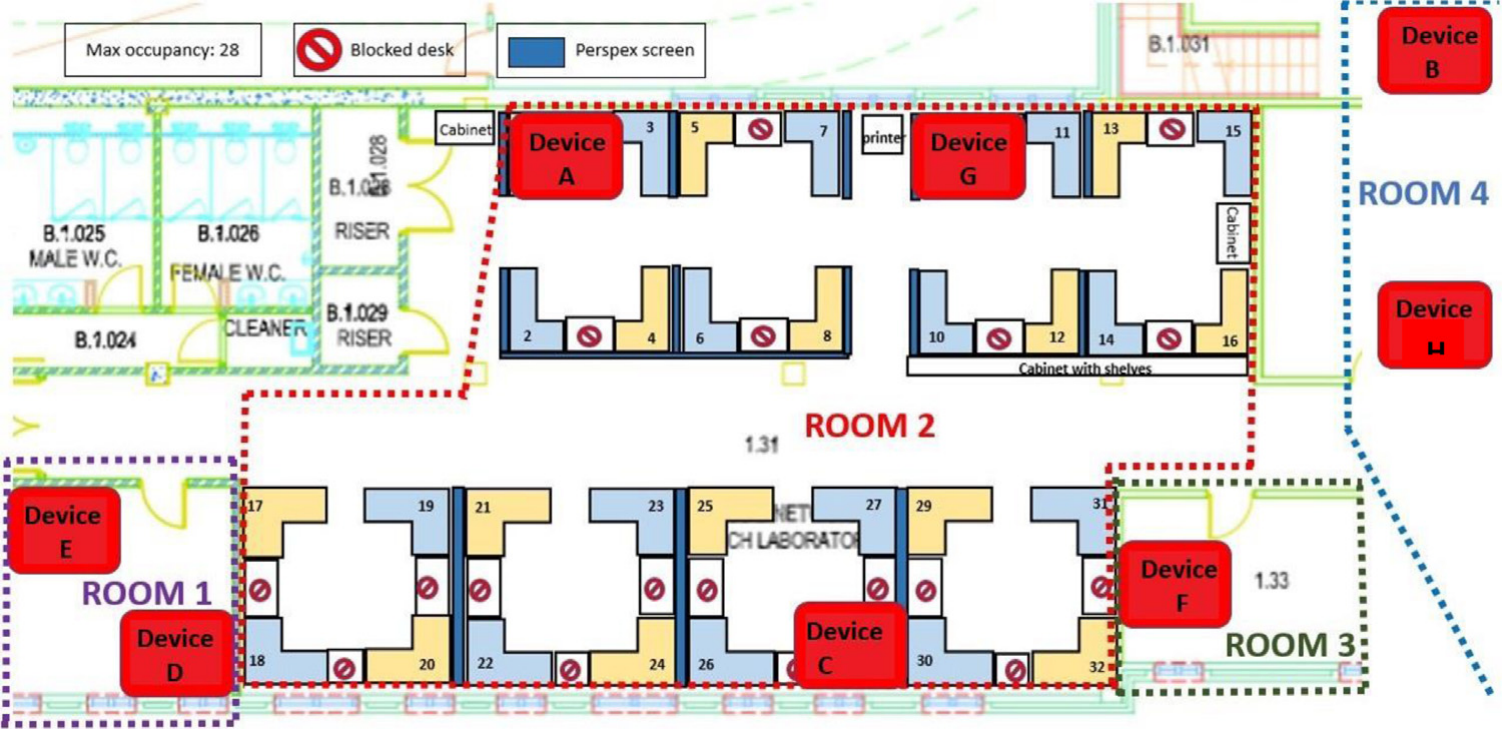
Communication Systems and Network Research Lab Plan and Node Locations

To collect real-world data, we have deployed an end-to-end IoT network in the University of Bristol, Communication Systems & Networks (CSN) Research Lab. The lab is actively used by a significant number of academic personnel and students. The number of occupants changes per day between 0 and 28. It is located on the second floor thus, it gets exposed to environmental changes such as seasonal temperature, humidity and light fluctuations. Furthermore, the endpoints are in different locations in the lab as in Fig. 3 to collect varying data due to differentiation between the areas. The network consists of eight stationary severely resource-constrained IoT endpoints, an additional device acting as the “edge'', and a server for data collection and controlling the experiment. Each IoT endpoint hosts sensors providing temperature, humidity, pressure, gas, accelerometer, and light readings. We collected two additional pieces of information: the measurements' accuracy value, calculated by the environmental sensors and the received signal strength indicator(RSSI) [9]. The measurements are sampled periodically, every 10 seconds, and sent from the endpoints to the edge device. The experiment started in February 2022, collecting, so far. We provide data which was collected until September 2022 over six-month period. We stored the sensor readings in the server cloud in CSV file format via an application we developed. In our analysis, only four sensor readings were used to illustrate our dataset (gas, humidity, temperature, and light). We also provide the histograms and time series of the dataset to show the distribution of sensor readings as can be seen in Fig. 1 and Fig. 2.

Each endpoint device of the network is a data collecting unit and consists of a Nordic nRF52840 DK board [10] and the following sensors:

(1) “ISL29125” Light Sensors: Collects intensity of the light as in Fig. 5.
(2) “MMA8452Q” Accelerometer Sensors as in Fig. 5.
(3) “BME680” Environmental Digital Sensors: Comprise of gas
  (VOC/CO2), pressure, temperature and humidity sensors as in Fig. 5.

**Fig. 4:**
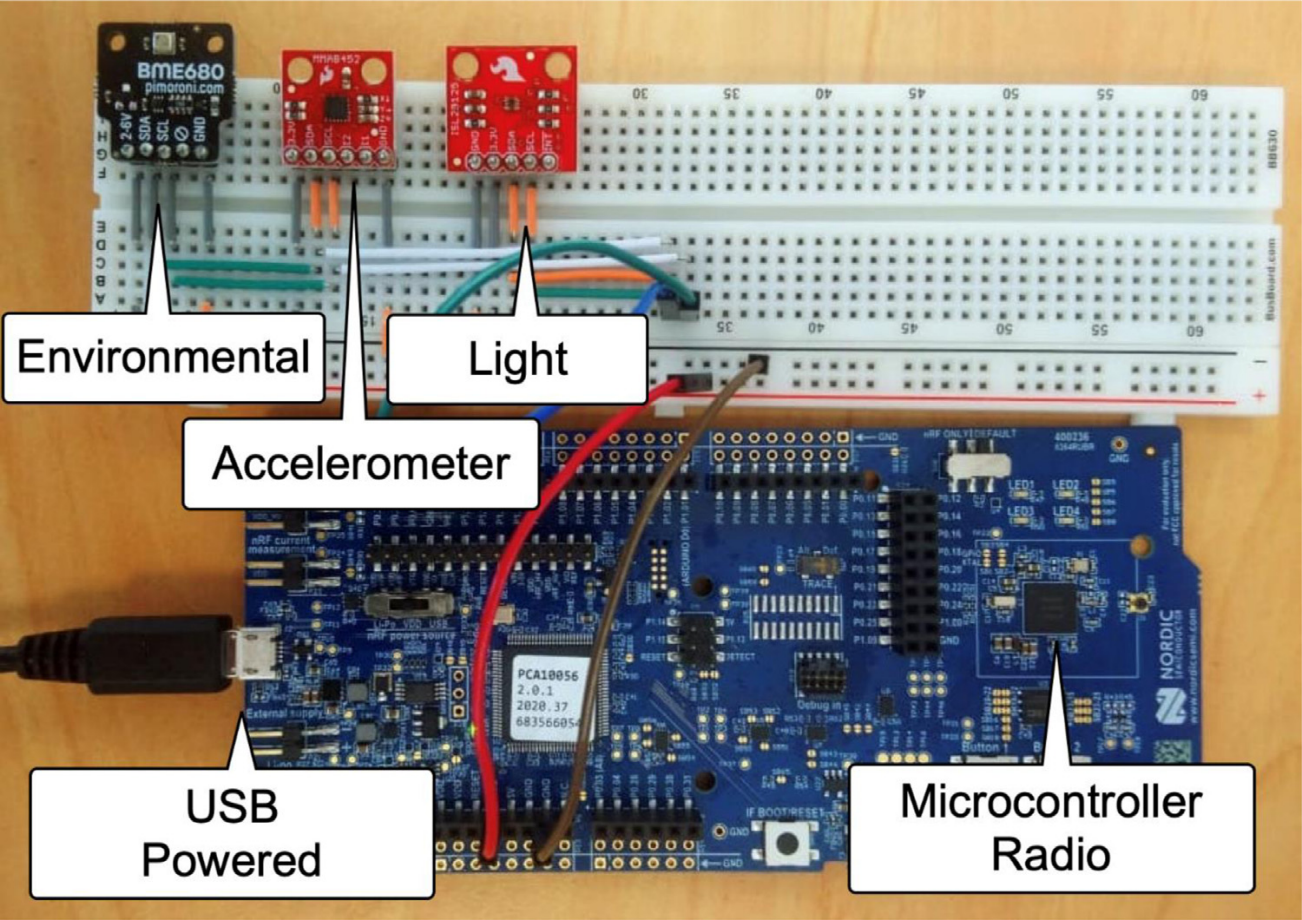
Endpoint with sensors.

The endpoint is identified using both the MAC address and a unique identifier provided by the vendor of the DK board. To easily locate every sensor deployed in the network, a map of the devices has been created, as shown in Fig. 4, where we report only the last digits of the identifiers. In case of failure of a device, we can easily find it in the office rooms.

**Fig. 5:**
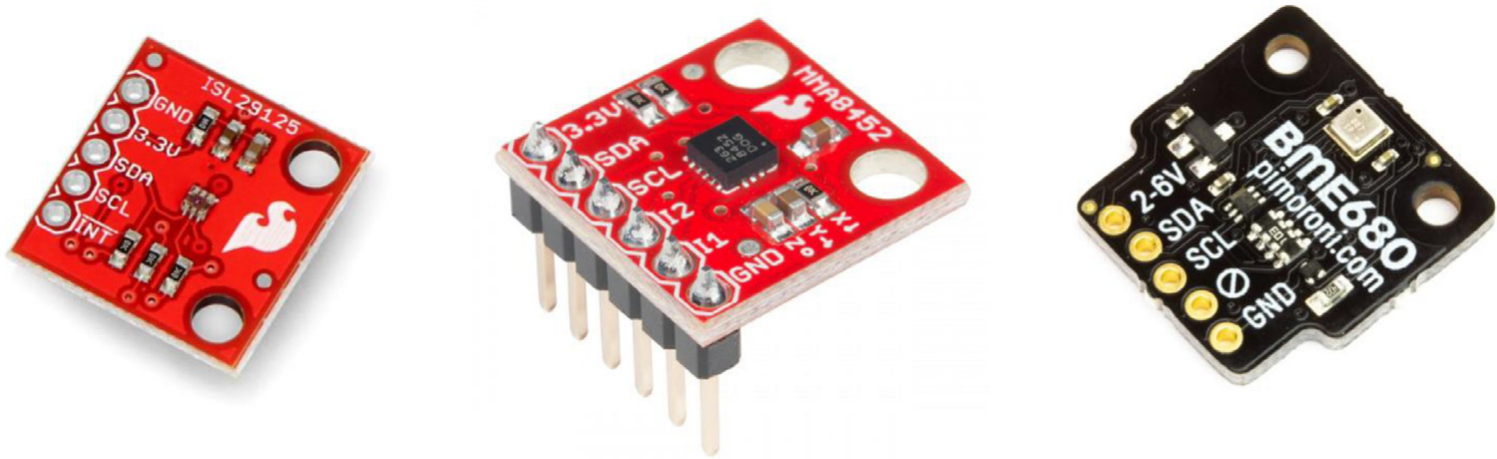
“ISL29125” light sensor, “MMA8452Q” accelerometer sensor and BME680 environmental digital sensor.

The DK board and the sensors are connected to every endpoint device using a breadboard. Communication is implemented using the I2C interface, where the DK board acts as a master and the three sensors act as slaves.

The endpoints are connected in a mesh topology, where the destination of the endpoints' data traffic is a device acting as the edge of the network. To enable communication between the endpoints and the edge of the network, we deployed, on the endpoints' DK board, the Contiki-NG operating system [11]. This provides a full stack implementation for forming mesh networks using IEEE 802.15.4 Time Slotted Channel Hopping (TSCH) MAC protocol [12], an IPv6 network layer and a UDP transport layer. The adoption of TSCH provides an effective solution to avoid interference and obtain healthy continuous data as shown in Fig. 7.

**Fig. 6:**
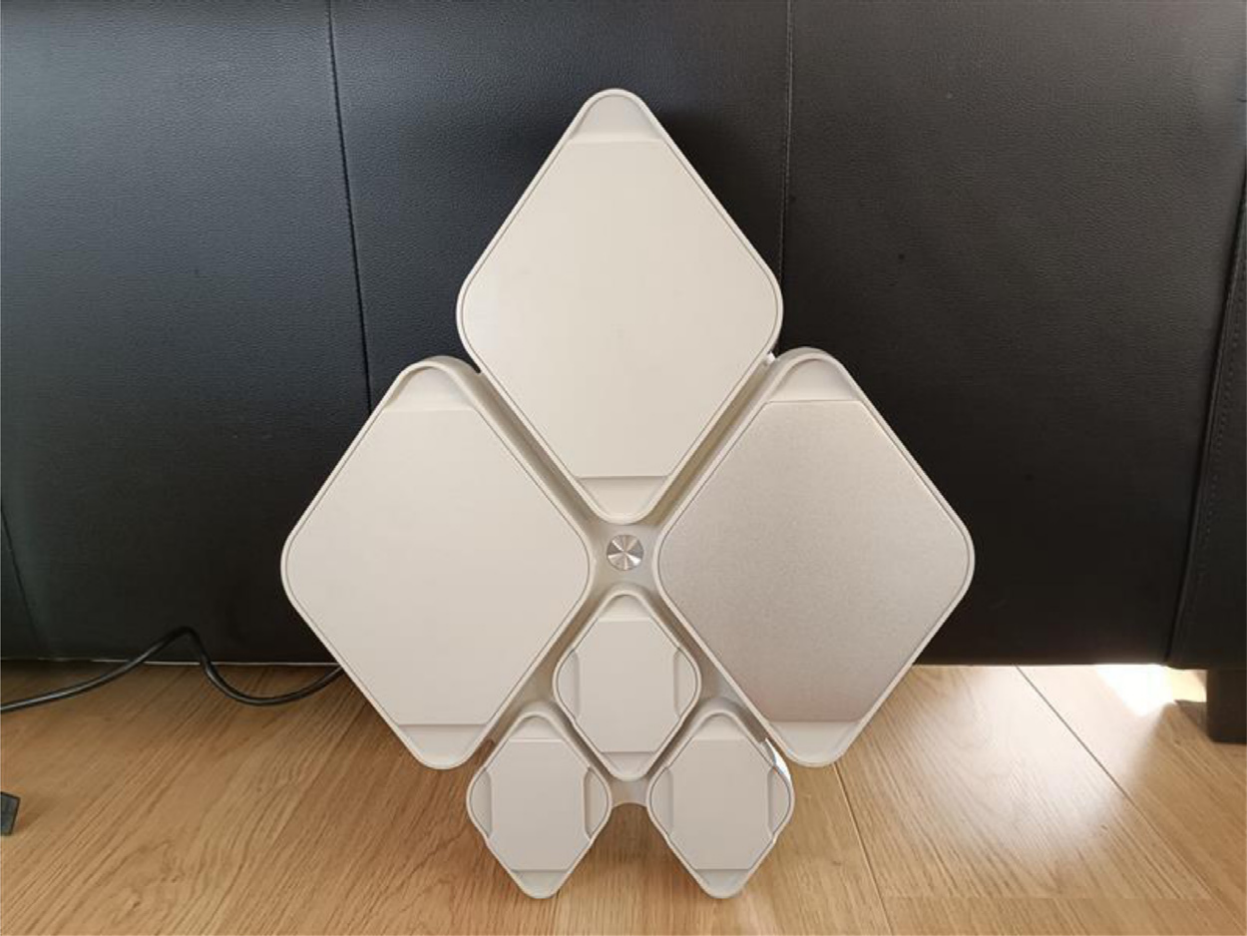
Umbrella edge node.

The device used as the edge of the mesh network is an UMBRELLA edge [5], equipped with a Nordic nRF52840 SoC and a Raspberry Pi as illustrated in Fig. 6. The Contiki-ng border router implementation has been deployed on the nRF52840 SoC. In this configuration, data is received by the nRF52840 SoC, using the IEEE 802.15.4 communication standard, and transferred to the Raspberry Pi.

**Fig. 7:**
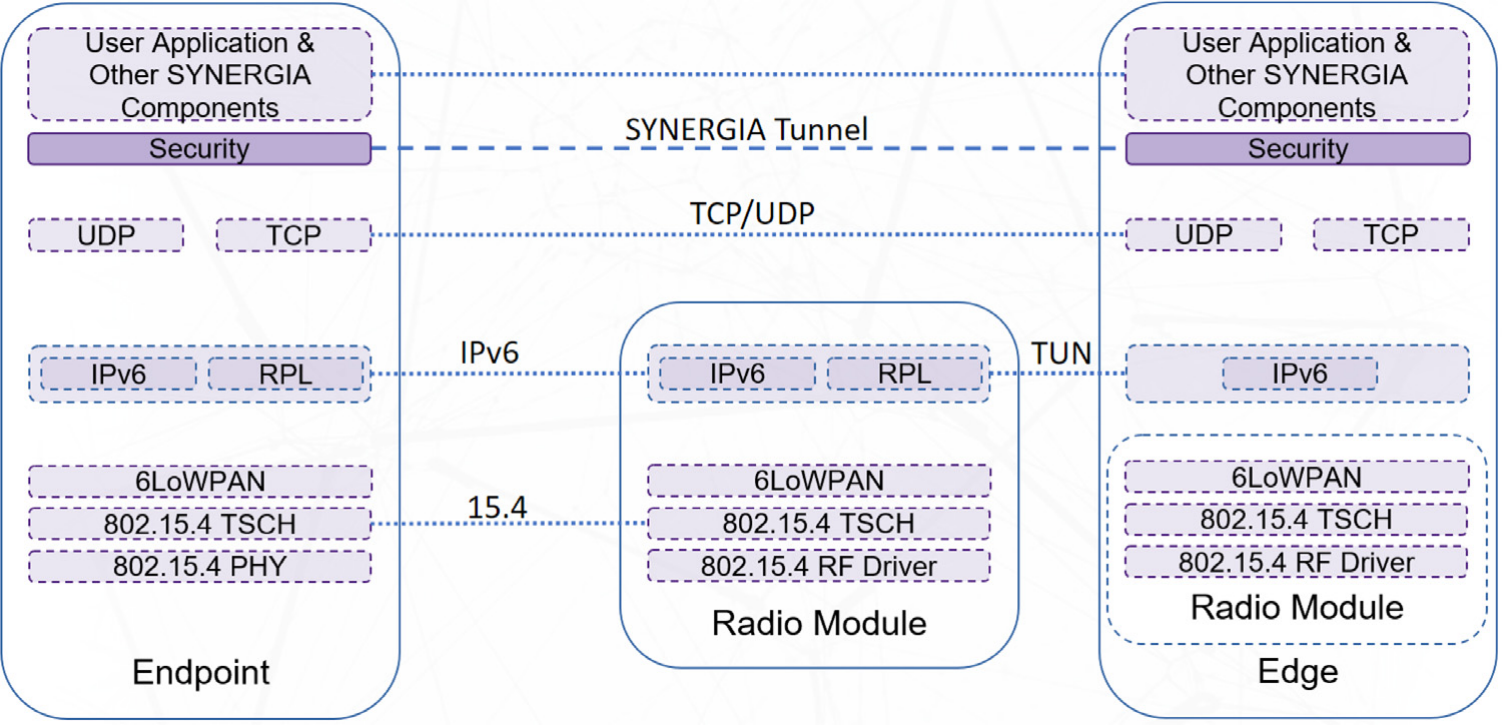
SYNERGIA infrastructure.

## 4 Experiment Control and Monitoring

On the Raspberry Pi acting as edge, we execute a series of software services, implemented in Python language, providing three functions:

1) Control of the experiment
2) Monitoring of the experiment
3) Data file format and storage

The control function communicates with the connected nRF52840 SoC, extracting the data originated by the endpoints. The monitoring functions verify that all the endpoints are sending sensor data correctly. The detection of an endpoint failure will be reported, providing the date of the failure and the identifier of the endpoint. Finally, the data file format and storage function are responsible for writing the received data in text files, using a Comma-Separated Values (CSV) format. Moreover, the files are periodically transferred to the server, so that they can be accessible via a Cloud service.

## Ethics Statements

The devices were deployed in a university lab space. Access to the area was limited to university students and faculty members. Care was taken to protect individual privacy. The data collection experiment was authorised in accordance with University of Bristol's research ethics approvals processes (application reference 10145). The dataset does not contain any personally identifiable information.

## CRediT Author Statement

**Ufuk Erol:** Manuscript Authoring, Deputy Data Steward, Lab Setup, Data Collection, Data Analysis; **Francesco Raimondo:** Manuscript Authoring, Lab Setup, Data Collection, Data Analysis; **James Pope:** Manuscript Authoring, Lab Setup, Data Collection, Data Analysis; **Samuel Gunner:** Lab Setup, Data Collection, Data Analysis; **Vijay Kumar:** Data Analysis; **Ioannis Mavromatis :** Manuscript Authoring, Data Analysis; **Pietro Carnelli:** Data Analysis; **Theodoros Spyridopoulos:** Data Analysis, Manuscript Authoring; **Aftab Khan:** Data Analysis; **George Oikonomou:** Data Steward, Manuscript Authoring, Data Collection, Data Analysis

## Declaration of Competing Interest

The authors declare that they have no known competing financial interests or personal relationships that could have appeared to influence the work reported in this paper.

## Data Availability

Multi-sensor, Multi-device Smart Building Indoor Environmental Dataset (Original data) (University of Bristol Data.Bris Research Data Repository).

## References

[1] SparkFun, Digital red, green and blue color light sensor with IR blocking filter, ISL29125, Sparkfun, 2014. https://cdn.sparkfun.com/datasheets/Sensors/LightImaging/isl29125.pdf.

[2] Semiconductors N. (2016). MMA8452Q, 3-axis, 12-bit/8-bit digital accelerometer. NXP Semicond..

[3] BOSCH, BME680 low power gas, pressure, temperature & humidity sensor, 1.8, 2022. https://www.bosch-sensortec.com/media/boschsensortec/downloads/datasheets/bst-bme680-ds001.pdf.

[4] OASIS, MQTT: the Standard for IoT messaging, n.d. https://mqtt.org/.

[5] BRIL Toshiba Europe Ltd., “UMBRELLA platform and testbed,” https://www.umbrellaiot.com, 2022, Accessed: 2022-1-31.

[6] Chimamiwa G., Alirezaie M., Pecora F., Loutfi A. (2021). Multi-sensor dataset of human activities in a smart home environment. Data Br..

[7] Luque Sánchez F., Hupont I., Tabik S., Herrera F. (2020). Revisiting crowd behaviour analysis through deep learning: taxonomy, anomaly detection, crowd emotions, datasets, opportunities and prospects. Inf. Fusion..

[8] Arifoglu D., Bouchachia A. (2019). Detection of abnormal behaviour for dementia sufferers using Convolutional Neural Networks. Artif. Intell. Med..

[9] Wu R.-H., Lee Y.-H., Tseng H.-W., Jan Y.-G., Chuang M.-H. (2008). 2008 IEEE Int. Conf. Ind. Technol..

[10] Nordic Semiconductor, nRF52840, v1.7, Nordic semiconductor, 2021. https://infocenter.nordicsemi.com/index.jsp?topic=%2Fug_nrf52840_dk%2FUG%2Fdk%2Fintro.html.

[11] Oikonomou G., Duquennoy S., Elsts A., Eriksson J., Tanaka Y., Tsiftes N. (2022). The Contiki-NG open source operating system for next generation IoT devices. SoftwareX.

[12] IEEE, IEEE Standard for low-rate wireless networks, IEEE Std 802.15.4-2015 (Revision IEEE Std 802.15.4-2011). (2016) 1–709.

[13] I. Mavromatis, A. Sanchez-Mompo, F. Raimondo, J. Pope, M. Bullo, I. Weeks, V. Kumar, P. Carnelli, G. Oikonomou, T. Spyridopoulos, & A. Khan, (Accepted/In press). LE3D: a lightweight ensemble framework of data drift detectors for resource-constrained devices. Paper presented at IEEE Consumer Communications & Networking Conference, Las Vegas, Nevada, United States.

